# Detection of Specific Immune Cell Subpopulation Changes Associated with Systemic Immune Inflammation–Index Level in Germ Cell Tumors

**DOI:** 10.3390/life12050678

**Published:** 2022-05-02

**Authors:** Katarina Kalavska, Zuzana Sestakova, Andrea Mlcakova, Paulina Gronesova, Viera Miskovska, Katarina Rejlekova, Daniela Svetlovska, Zuzana Sycova-Mila, Jana Obertova, Patrik Palacka, Jozef Mardiak, Miroslav Chovanec, Michal Chovanec, Michal Mego

**Affiliations:** 1 Translational Research Unit, Faculty of Medicine, Comenius University, 833 10 Bratislava, Slovakia; katarina.kalavska@nou.sk (K.K.); daniela.svetlovska@nou.sk (D.S.); 2 Department of Molecular Oncology, Cancer Research Institute, Biomedical Research Center, Slovak Academy Sciences, 814 39 Bratislava, Slovakia; 3 Department of Genetics, Cancer Research Institute, Biomedical Research Center, Slovak Academy Sciences, 814 39 Bratislava, Slovakia; zuzana.sestakova@savba.sk (Z.S.); miroslav.chovanec@savba.sk (M.C.); 4 Department of Laboratory Medicine, National Institute of Children’s Diseases, 831 01 Bratislava, Slovakia; 5 Department of Hematology, National Cancer Institute, 833 10 Bratislava, Slovakia; andrea.mlcakova@nou.sk; 6 Department of Tumor Immunology, Cancer Research Institute, Biomedical Research Center, Slovak Academy Sciences, 814 39 Bratislava, Slovakia; paulina.gronesova@savba.sk; 7 1st Department of Oncology, Faculty of Medicine, St. Elisabeth Cancer Institute, Comenius University, 812 50 Bratislava, Slovakia; vieramiskovska@ousa.sk; 8 2nd Department of Oncology, Faculty of Medicine, National Cancer Institute, Comenius University, 833 10 Bratislava, Slovakia; katarina.rejlekova@nou.sk (K.R.); jana.obertova@nou.sk (J.O.); patrik.palacka@nou.sk (P.P.); jozef.mardiak@nou.sk (J.M.); michal.chovanec1@nou.sk (M.C.); 9 Department of Oncology, National Cancer Institute, 833 10 Bratislava, Slovakia; zuzana.sycova-mila@nou.sk

**Keywords:** germ cell tumors, systemic immune–inflammation index, leukocyte subpopulations, neutrophilia, lymphocytopenia

## Abstract

The tumor microenvironment (TME) and the host inflammatory response are closely interconnected. The interplay between systemic inflammation and the local immune response may influence tumor development and progression in various types of cancer. The systemic immune–inflammation index (SII) represents a prognostic marker for germ cell tumors (GCTs). The aim of the present study was to detect specific immune cell subpopulation changes which were associated with the SII level in chemotherapy-naïve GCT patients. In total, 51 GCT patients, prior to cisplatin-based chemotherapy, were included in the present study. Immunophenotyping of peripheral blood leukocyte subpopulations was performed using flow cytometry. The SII level was correlated with the percentage of various leukocyte subpopulations. The obtained results demonstrated that SII levels above the cut-off value of SII ≥ 1003 were associated with higher neutrophil percentages. An inverse correlation was found between the SII and the peripheral lymphocyte percentage that logically reflects the calculations of the SII index. Furthermore, the presented data also showed that in the lymphocyte subpopulation, the association with the SII was driven by T-cell subpopulations. In innate immunity–cell subpopulations, we observed a correlation between SII level and neutrophils as well as associations with eosinophil, basophil, natural killer cell and dendritic cell percentages. We suppose that the described interactions represent a manifestation of cancer-induced immune suppression. The results of the present study contribute to the elucidation of the interrelationship between tumor cells and the innate/adaptive immune system of the host.

## 1. Introduction

The tumor microenvironment (TME) and the host inflammatory response are closely interconnected. Increasing evidence suggests that the interplay between systemic inflammation and the local immune response may influence tumor development and progression in various types of cancer [1,2,3,4]. Moreover, inflammation is reported to be a mechanism that participates in tumor immunoresistance [2]. The ‘complete blood count’ represents a simple tool for monitoring systemic inflammation via immune–inflammatory elements, including neutrophils, lymphocytes and platelets, which may help characterize the inflammatory TME [5,6]. In addition to these aforementioned single-parameter markers, several inflammatory cell ratios derived from these mediators, such as the neutrophil/lymphocyte ratio (NLR), platelet/lymphocyte ratio (PLR), C-reactive protein/albumin ratio and systemic immune–inflammation index (SII) have previously been investigated. These markers consider several different types of immune cells characterized by both pro-tumor and anti-tumor activities, and therefore more accurately reflect the host inflammatory response to tumor cells [7].

The SII is assessed using peripheral neutrophil, lymphocyte and platelet counts and was first described by Hu et al. [8]. Therefore, the SII is considered to reflect the interplay between the local immune response and systemic inflammation in patients with cancer [9,10,11]. Neutrophils are able to secrete several inflammatory mediators, including vascular endothelial growth factor, IL-6, IL-10 and IL-22 and are therefore implicated in tumor-promoting activities [12]. Platelets have previously been reported as immune cells that support the formation of metastases and disease spread via the promotion of tumor cell arrest in the endothelium [13]. Platelets have also been reported to protect cancer cells from lysis by natural killer (NK) cells [14]. Moreover, lymphocytes inhibit tumor burden, and therefore their low levels may lead to an inadequate immunological response in patients with cancer [15].

The SII can also be used as a prognostic marker, predicting a patient’s survival in numerous types of cancer [4,16,17,18,19,20,21]. Moreover, recent data indicates that the SII is of higher prognostic value compared with other biomarker ratios, such as the NLR and PLR [16,22,23,24,25].

Numerous reports on germ cell tumors (GCTs) have also demonstrated the prognostic value of SII [7,26,27]. Chovanec et al. reported that the SII is significantly associated with poor-risk clinical features. Low SII levels determined prior to chemotherapy are correlated with longer progression-free survival (PFS) and overall survival (OS) [26]. Similar results were obtained in a study of 146 patients with metastatic GCTs undergoing first-line chemotherapy, whereby a higher SII was independent of the International Germ Cell Cancer Collaborative Group (IGCCCG) risk classification associated with a worse OS [7]. Göger et al. compared the SII levels in testicular cancer patients with a control group: the median SII was demonstrated to be significantly higher in testicular tumors. Furthermore, subgroup analysis revealed that the median SII value was significantly higher in testicular cancer patients with advanced disease stages and the presence of metastases. The SII value was also confirmed as an independent predictor of OS in this cohort of patients [27]. Furthermore, the predictive role of SII was assessed by Cursano et al. in a study involving 62 GCT patients receiving high-dose chemotherapy (HDCT). The results from this study suggested that SII is significantly associated with the overall response to HDCT as well as with patient outcome [28].

Testicular cancer is the most common type of solid tumor in 20–40-year-old males. The incidence of this disease is increasing worldwide. GCTs are traditionally referred to as chemotherapy-sensitive malignancies, with a 5-year survival rate of 98% for localized disease [29,30]. The mammalian testes are characterized as immunologically privileged sites, where a balance between immune privilege and the ability to respond to infections and inflammation plays an important role [31]. However, knowledge of immune cell roles in GCTs is rather limited. Siska et al. reported that advanced stages of testicular tumors are associated with an increased signature of regulatory T cells, neutrophils and mast cells, regardless of the histological subtype. Moreover, elevated levels of macrophage signatures and decreased T-cell and NK-cell signatures are also detected [32].

The aim of the present study was to identify the specific subpopulations of innate and adaptive immune cells that are associated with changes in the SII levels in chemotherapy-naïve GCTs. To achieve this, the percentage of immune cell subpopulations was associated with SII.

## 2. Materials and Methods

### 2.1. Patients

In total, 51 chemotherapy-naïve GCT patients, treated between January 2017 and January 2020 at the National Cancer Institute (Bratislava, Slovakia), and with available SII and immunophenotype data, were enrolled in the present study. Patients with evidence of concomitant malignancies, with the exception of nonmelanoma skin cancer, in the previous 5 years were excluded from the study. The following clinical data was collected for each participant: age, tumor histologic subtype, clinical stage of primary disease at diagnosis, type and number of metastatic sites and the date of diagnosis. The clinical stage of the primary disease was determined according to the tumor–node–metastasis staging system 2017 criteria. TNM classification combines the anatomic extent of disease (including primary tumor (pT), regional lymph nodes (pN) and distant metastasis (pM)) with the serum tumor markers alpha-fetoprotein (AFP), human chorionic gonadotropin (hCG) and lactate dehydrogenase (LDH), which are surrogates for anatomic disease extent [33]. The response to the therapy was assessed among the evaluable population according to standard RECIST (Response Evaluation Criteria In Solid Tumors) criteria, version 1.1 [34]. Favorable response to therapy included patients with complete remission or partial remission with negative tumor markers. The Institutional Review Board and Ethical Committee of the National Cancer Institute, Bratislava, Slovakia (protocol no. IZLO1; Chair: M. Mego, from 10 February 2010) approved the study protocol. A written informed consent form was signed by each participant prior to enrollment into the study.

### 2.2. SII

The SII was determined using counts of peripheral blood platelets (P), neutrophils (N) and lymphocytes (L) per liter, which were retrieved from routine prechemotherapy blood tests. The equation SII = P × N/L was used. A cut-off value of SII ≥ 1003 was chosen in order to dichotomize SII into low (<1003) and high (≥1003) categories according to the previous study by Chovanec et al. [26].

### 2.3. Determination of Leukocyte Immunophenotypes

In the morning of day −1 or 0 of first-line of chemotherapy, 1 mL atraumatic peripheral blood was collected at the antecubital fossa into an EDTA-treated collection tube. Analyzed samples were processed within 24 h following collection, as previously described by Kalavska et al. [34]. Briefly, leukocytes were stained using fluorochrome-conjugated antibodies from BD Pharmingen and, subsequently, leukocytes with defined immunophenotypes were quantified using flow cytometry (Canto II Cytometer; Becton, Dickinson and Company, Franklin Lakes, NJ, USA). The following antibody combinations were used for the basic panel: CD8 FITC (clone SK1, cat. no.: 345772, BD Biosciences, San Jose, CA 95131, USA), CD56 phycoerythrin (PE (clone MY31, cat. no.: 345810, BD Biosciences, San Jose, CA 95131, USA), CD45 PerCP Cy5.5 (clone SK3, cat. no.: 332772, BD Biosciences, San Jose, CA 95131, USA), CD19 PE-Cy7 (cat. no.: IM3628, Beckman Coulter Immunotech SAS, Marseille, France), CD3 APC (clone SK7, cat. no.: 345767, BD Biosciences, San Jose, CA 95131, USA), CD16 APC-H7 (clone 3G8, cat. no.: 560195, BD Pharmingen, San Diego, CA 92121, USA), CD4 V450 (clone RPA-T4, cat. no.: 560345, BD Biosciences, San Jose, CA 95131, USA) and CD14 HV500 (clone M5E2, cat. no.: 561391, BD Biosciences, San Jose, CA 95131, USA). The regulatory T-cell panel included the following antibodies: CD3 FITC (clone SK7, cat. no.: 345763, BD Biosciences, San Jose, CA 95131, USA), CD127 PE (clone hIL-7R-M21, cat. no.: 557938, BD Pharmingen, San Diego, CA 92121, USA), CD4 PerCP Cy5.5 (clone SK3, cat. no.: 566923, BD Biosciences, San Jose, CA 95131, USA), CD25 PE-Cy7 (clone 2A3, cat. no.: 335824, BD Biosciences, San Jose, CA 95131, USA) and CD45 HV450 antibody (clone HI30, cat. no.: 560367, BD Biosciences, San Jose, CA 95131, USA). For the dendritic-cell (DC) panel, the following antibodies were used: Lineage cocktail 2 FITC (cat. no.: 643397, BD Biosciences, San Jose, CA 95131, USA), CD1c PE (clone F10/21A3, cat. no.: 564900, BD Pharmingen, San Diego, CA 92121, USA), human leukocyte antigen (HLA)-DR PerCP (clone, L243, cat. no.: 347402, BD Biosciences, San Jose, CA 95131, USA), CD123 PE-Cy7 (clone 7G3, cat. no.: 560826, BD Pharmingen San Diego, CA 92121, USA), CD11c APC (clone B-Ly 6, cat. no.: 560895, BD Biosciences, San Jose, CA 95131, USA), CD16 APC-H7 (clone 3G8, cat. no.: 560195, BD Pharmingen, San Diego, CA 92121, USA) and CD45 HV450 (clone HI30, cat. no.: 560367, BD Biosciences, San Jose, CA 95131, USA) Finally, the myeloid-derived suppressor–cell panel used the following antibodies: CD15 FITC (cat. no.: IM1423U, Beckman Coulter Immunotech SAS, Marseille, France), CD11b PE (cat. no.: IM2581U, Beckman Coulter Coulter Immunotech SAS, Marseille, France), HLA-DR PerCP (clone L243, cat. no.: 347402, BD Biosciences, San Jose, CA 95131, USA), CD62L PE-Cy7 (clone DREG-56, cat. no.: 565535, BD Biosciences, San Jose, CA 95131, USA), CD33 APC (clone P67.6, cat. no.: 345800, BD Biosciences, San Jose, CA 95131, USA), CD14 APC-H7 (clone MΦP9, cat. no.: 641394, BD Biosciences, San Jose, CA 95131, USA), CD66b V450 (clone G10F5, cat. no.: 561649, BD Biosciences, San Jose, CA 95131, USA) and CD45 BV510 antibody (clone 30-F11, cat. no.: 103138, BioLegend, San Diego, CA 91121, USA). A cocktail of the aforementioned antibodies was incubated with 300,000–500,000 white blood cells in 200 µL for 20 min at room temperature. Before the fixation of cells using 1X BD FACS Lysing Solution (BD Bioscience, San Jose, CA, USA, cat. no: 349202), lysis of red blood cells was performed. For the assessment with a BD FACSCanto™ II flow cytometer (Becton Dickinson, Franklin Lakes, NJ, USA), a minimum of 100,000 leukocytes were utilized. KALUZA software (Beckman Coulter, Inc., Brea, CA, USA) was used for the analysis of the flow cytometry data. Forward scatter (FSC) and side scatter were used to exclude debris according to size and granularity, while exclusion of doublets was performed using FSC-Height and FSC-Area. The number of gated cells considered as the minimum for evaluation was 100.

### 2.4. Statistical Analysis

Patient data were tabulated and subsequently assessed using descriptive statistical methods. The median values (ranges) were used as continuous variables, whereas categorical variables were assessed by frequency (percentage). The distribution of the data was evaluated using the Kolmogorov–Smirnov test. Normally distributed data were assessed using one-way ANOVA, whereas non-normally distributed data were statistically analyzed using the nonparametric Mann–Whitney U test or Kruskal–Wallis H test. Pearson’s correlation coefficient or Spearman’s rank-correlation coefficient tests were used according to the normality of the data.

A cut-off value of SII ≥ 1003 was chosen in order to dichotomize SII into low (<1003) and high (≥1003) categories according to the previous study by Chovanec et al. [26]. The used cut-off of ≥1003 represents, as previously reported in this work, the median value of SII obtained from the discovery set of GCT patients, which was subsequently verified in survival analyses performed on an independent validation GCT patients cohort. The dichotomized data were further associated with specific immune cell counts using univariate analysis. Subsequently, a multivariate logistic regression analysis was performed, which included the variables identified as significantly linked to the SII level in the univariate analysis.

Similarly, the data dichotomized as ‘low’ or ‘high’ according to the SII cut-off value were used in survival analyses. The median follow-up period was defined as the median observation time among all patients and among the patients who were alive at the time of their last follow-up. PFS was defined as the period from day 1 of the first cycle of chemotherapy administration to the date of the progression of the disease or last follow-up, whereas OS was defined as the time from day 1 of the first cycle of chemotherapy administration to the date of death or last follow-up. PFS and OS were assessed using the Kaplan–Meier product-limit method and were compared between different groups using the log-rank test. Hazard ratios (HRs) and 95% confidence intervals (CIs) were estimated using logistic regression and Cox proportional hazard analysis, respectively.

Statistical analysis was performed using NCSS 11 Statistical Software (NCSS, LLC., Kaysville, UT, USA, ncss.com/software/ncss, accessed on 4 December 2021). Data are presented as the mean ± SEM. All the presented *p*-values are two-sided. A value of *p* ≤ 0.05 was considered to indicate a statistically significant difference.

## 3. Results

### 3.1. Patient Characteristics

In total, 51 chemotherapy-naïve GCT patients were enrolled in the present study. Patient baseline characteristics are summarized in Table 1. The median patient age was 34 years (range, 22–59 years). The majority of patients (62.7%) were classified in a good risk group according to the IGCCCG criteria. Primary tumors were predominantly located in the testes (48 patients; 94.1%). Overall, 39 patients (76.5%) presented with nonseminomatous histology, whereas 12 patients (23.5%) had seminomatous GCTs. Most patients (56.9%) had metastatic disease with one to two metastatic sites. Metastases were located mainly in retroperitoneal lymph nodes (74.5%). Platinum-based chemotherapy was administrated to all enrolled patients. All patients also received granulocyte colony–stimulating factor support (filgrastim or pegfilgrastim) following chemotherapy.

### 3.2. Correlation between the SII Level and Percentage of Different Innate Immune Cells in Chemotherapy-Naïve GCT Patients

Using univariate logistic regression analysis, the results demonstrated that patients with an SII under the cut-off value (≥1003) had a significantly lower mean percentage of neutrophils ± standard error of the mean (SEM) 56.7 ± 1.7%, compared with patients with an SII higher than the cut-off value, 74.5 ± 2.2% (*p* < 0.00001). Similarly, a lower NK cell percentage correlated with an SII under the cut-off value (10.3 ± 1.6% vs. 18.3 ± 2.1%; *p* = 0.02264). Moreover, a negative correlation was detected between an SII under the cut-off value and a higher percentage of eosinophils (3.3 ± 0.4% vs. 1.4 ± 0.5%; *p* = 0.00431) and basophils (0.7 ± 0.06% vs. 0.5 ± 0.07%; *p* = 0.00852). Moreover, a higher mean percentage of DCs (0.9 ± 0.08% vs. 0.7 ± 0.11%; *p* = 0.02728), as well as plasmocytoid DCs (0.2 ± 0.01% vs. 0.1 ± 0.02%; *p* = 0.00310), significantly correlated with an SII level under the cut-off value. However, statistical analysis of the association between the SII and innate immune cell–count using multivariate logistic regression analysis demonstrated that only the percentage of neutrophils was independently associated with the SII level, dichotomized according to the cut-off value (Table 2).

### 3.3. Association between the SII Level and Selected Adaptive Immune Cell Percentages

It was determined using univariate logistic regression analysis that a higher percentage (±SEM) of lymphocytes was significantly associated with a low SII level (31.2 ± 1.5% vs. 15.0 ± 2.0%; *p* < 0.00001). A similar association was also demonstrated between T-cell and cytotoxic T-cell percentages and the SII. In patients with a low SII, a significantly higher percentage of T cells was determined compared with the patients with a high SII (76.3 ± 1.7% vs. 68.1 ± 2.2%; *p* = 0.01410). Furthermore, a higher percentage of cytotoxic T cells was observed in patients with a low SII (27.7 ± 1.0% vs. 23.4 ± 1.3%; *p* = 0.02383).

Multivariate analysis of all adaptive immune-cell subpopulations that were significantly linked to the SII level in the univariate analysis determined that CD3+ T cells were the only subpopulation of adaptive immune cells that were independently associated with the SII level in GCT patients (*p* = 0.01385) (Table 3).

### 3.4. The Prognostic Role of the SII

**Figure 1:**
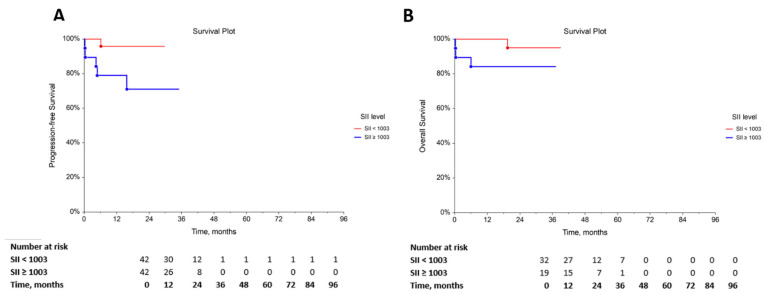
Kaplan–Meier curves. The probabilities of (**A**) PFS (*p* = 0.0274) and (**B**) OS (*p* = 0.1025) according to the SII value.

The determination of PFS and OS according to the SII was estimated using Kaplan–Meier analysis in GCT patients. The cut-off value of SII used in this analysis was ≥1003, according to our previous study [26]. The results demonstrated that patients with a low SII had a significantly longer PFS (HR, 0.13; 95% CI, 0.03–0.67; *p* = 0.0274; Figure 1A), whereas the prognostic value of SII in determining OS was not statistically significant (HR, 0.19; 95% CI, 0.02–1.44; *p* = 0.1025; Figure 1B).

For each time interval, the survival probability, expressed as number at risk, was calculated as the number of subjects surviving divided by the number of patients at risk. Subjects who died or dropped out were not counted as “at risk”.

## 4. Discussion

Growing evidence suggests there is an intensive cross-talk between the host immune system and cancer. Inflammation is implicated in several aspects of cancer biology, including cancer development, progression and prognosis [5,35,36]. The malignant process influences the host immune system not only at the tumor site but also on a global level by forming a systemic inflammatory response [37]. An impaired intratumoral inflammatory response and an elevated systemic response suggests decreased immunological control of tumors at the local level, whereas the formation of a systemic pro-inflammatory environment creates suitable conditions for cancer progression [38,39]. Therefore, deeper insight into both the innate and adaptive immune system responses within the TME may lead to a better understanding of the interactions between cancer and host immune cells.

The aim of the present study was to determine which subpopulations of innate and adaptive immune cells are associated with SII in GCT patients. To the best of our knowledge, this is the first study to assess the link between the peripheral immune cells and the SII prior to chemotherapy. The results demonstrated a positive correlation between the SII level and neutrophil percentage, whereas an inverse association was determined between the SII value and T-cell percentage.

Recently, an increasing number of studies have reported that SII may serve as a prognostic marker in GCTs, whereby high SII levels are significantly associated with worse PFS and OS [7,26,27]. In the present study, the prognostic value of SII was also confirmed in GCT patients. A significant association between PFS and the SII value was reported. The association between SII and OS was not statistically significant, which may be explained by the limited number of patients enrolled in the present study. A strong significant association between SII and poor clinical features, such as primary extragonadal tumors, bulky retroperitoneal disease, nonpulmonary visceral metastases and elevated tumor markers was also observed. This data suggest that the host immune system is implicated in the progression of GCTs. However, the results of the present study did not determine whether systemic inflammation, expressed by the SII, formed a permissive microenvironment which resulted in disease characterized by poor clinical features, or whether the SII simply reflected an aggressive disease [26].

The SII is a value determined by a combination of the following three parameters: (i) neutrophils, (ii) platelets and (iii) lymphocytes. Therefore, a high SII could be attributed to changes in the counts of these cells [40]. Ma et al. reported that high pretreatment levels of SII reflect increased neutrophil and platelet counts or decreased lymphocyte counts [41]. Results obtained in the present study are in accordance with these data. The results demonstrated that a high SII level was independently associated with elevated peripheral neutrophils and decreased lymphocytes simultaneously. Neutrophilia is relatively common in patients with cancer. Neutrophils contribute to the creation of a highly immunosuppressive microenvironment via numerous signaling pathways, and therefore neutrophilia facilitates tumor growth and metastasis [42]. Low lymphocyte counts are traditionally considered a reflection of impaired host immunosurveillance. Lymphocytopenia observed prior to treatment could be regarded as a surrogate marker of cancer-induced immunosuppression and its prognostic role has previously been described in several solid types of cancer, including metastatic breast, renal and colorectal cancer [43,44]. However, the causes of tumor-induced lymphocytopenia are not fully understood. Recent data postulates that low peripheral lymphocyte counts are a consequence of impaired lymphocyte homeostasis and the increased activation of lymphocyte apoptosis [43]. This is a result of the enhanced secretion of several immunosuppressive molecules, including TGF-β, by the tumor and others factors in the TME, which lead to the impairment of cytotoxic and helper lymphocyte activation, whereas the recruitment of suppressive regulatory T cells is promoted [45]. It can also be hypothesized that there are common signaling pathways implicated in the immune escape of malignant cells which simultaneously support lymphocytopenia in the host immune system [43].

Beyond the logical correlation between the neutrophil percentage and SII level, the present study also determined the association of SII with other innate immune cell subpopulations, including eosinophils, basophils, NK cells and DCs. Eosinophils, as well as lymphocytes, neutrophils and macrophages, are important factors in the cross-talk between inflammation and cancer [46]. Depending on the microenvironment and biological interactions [47,48], eosinophils may be implicated in both the pro-inflammatory and anti-inflammatory signaling pathways. Furthermore, the varying impacts of eosinophil count on a patient’s outcome are reported to depend on the type of malignancy. A high eosinophil count is correlated with a better prognosis in hepatocellular carcinoma treated with sorafenib, in melanoma, renal carcinoma, and in colorectal, lung, cervical and pancreatic cancer. However, eosinophilia is an unfavorable prognostic marker in breast cancer and lymphoma [49,50,51,52,53,54,55,56,57,58]. In the present study, the association between a low SII and eosinophilia in chemotherapy-naïve GCT patients was described.

A similar association was observed between the baseline basophil percentage and SII. Recent studies have demonstrated both a protective [59] and pro-tumorigenic role [60,61] of basophils in tumorigenesis. Numerous studies have reported that basophils affect the TME of human [61,62,63] and experimental [60,61] tumors. The connection between the peripheral blood basophils and tumor burden has been reported in certain solid tumors [64]. Basopenia is suggested to be a negative prognostic marker in patients with colorectal cancer [51,65]. Furthermore, basophilia is reported to be associated with a better outcome in patients with melanoma treated with immunotherapy (nivolumab with ipilimumab) [50]. Basophilia as a positive prognostic marker has also been demonstrated in patients with ovarian cancer [63].

An interesting association between NK cells and SII was revealed in the present study. A high NK-cell percentage was significantly correlated with a high SII. NK cells represent a specialized population of innate immune cells which play a critical role in the host immune response against tumor growth [66,67]. The importance of NK cells in tumor immunosurveillance and in the mediation of antimetastatic effects has previously been determined in mouse models and clinical studies [67,68,69,70]. High levels of tumor-infiltrating NK cells are associated with a good prognosis in certain solid tumors, such as breast cancer [71], gastrointestinal stromal tumors [72,73,74], neuroblastoma [75], head and neck cancer [76] and prostate cancer [77]. However, there are malignancies that are refractory to their antitumor function, mainly due to the presence of immunosuppressive microenvironment favoring neoplastic progression [66,67]. The results of the present study are in line with these observations as an immunosuppressive TME (expressed as a high SII) was associated with a high percentage of NK cells herein. A high SII level was confirmed as a negative prognostic marker for PFS. However, understanding the mechanism of how the TME is able to hinder NK-cell function remains to be elucidated.

The last subpopulation of innate immune cells that displayed percentage changes and was significantly associated with SII was represented by DCs. DCs are specialized antigen-presenting cells that are responsible for the initiation of specific T-cell responses and humoral responses which inhibit tumor development [78,79,80,81]. Several reports have demonstrated that the DC percentage in patients with cancer is significantly lower compared with healthy subjects [82,83,84,85]. Furthermore, the spontaneous apoptosis of peripheral DCs in patients with cancer has been described and is hypothesized to be the result of culture conditions or contact with cancer cells [86,87,88,89]. However, the role of pDCs in the TME remains controversial. While tumor-infiltrating pDCs possess immunosuppressive properties, the ability of pDCs to produce type I IFN and TNF-α indicates their antitumorigenic potential [90]. The human pDCs gene signature has been described as a positive prognostic factor in lung carcinoma [91], whereas the presence of pDCs in breast cancer is associated with a poor prognosis [92].

Assessing the changes in adaptive immune cell subpopulations in association with the SII level demonstrated the inverse correlation between cytotoxic T cells and the SII level. Cytotoxic T lymphocytes are considered to be the most powerful immune cells in the anticancer immune response [93]. However, during cancer progression, cytotoxic T cells display dysfunction and exhaustion as a consequence of immune-related tolerance and the presence of immunosuppression within the TME [94].

The present study has certain limitations related to the limited number of recruited patients. Furthermore, the competence or functions of selected leukocyte subpopulations were not analyzed. Therefore, a larger data set is needed in order to determine the characteristics of the selected leukocyte subpopulations associated with SII in GCT patients.

In conclusion, the present study demonstrates that high pretreatment SII levels are associated with higher neutrophil and lower lymphocyte percentages in the analyzed GCT patients. Beyond the association with neutrophil and lymphocyte percentages that are involved in the SII assessment, it was also observed that this association was driven by the T-cell subpopulation. Furthermore, by assessing innate immune cells, we showed that, beyond the correlation between neutrophil percentage and SII level, correlations with eosinophil, basophil, NK cell and DC percentages were also determined.

## Figures and Tables

**Table 1:** Patient characteristics.

Variable	*N*	%
All patients	51	100.0
Histology		
Seminoma	12	23.5
Nonseminoma	39	76.5
Primary tumor localization		
Testicular	48	94.1
Extragonadal	3	5.9
IGCCCG risk group		
Good risk	33	64.7
Intermediate risk	5	9.8
Poor risk	13	25.5
Stage IA and IB (adjuvant therapy)	9	17.6
Sites of metastases		
Retroperitoneum	38	74.5
Mediastinum	7	13.7
Lungs	15	29.4
Liver	7	13.7
Brain	2	3.9
Other	1	2.0
Visceral nonpulmonary metastases	9	17.6
No. of metastatic site(s)		
0	10	19.6
1 to 2	29	56.9
>3	12	23.5
Staging (UICC)		
IA	2	3.9
IB	7	13.7
IS	1	2.0
IIA	4	7.8
IIB	9	17.6
IIC	1	2.0
IIIA	7	13.7
IIIB	6	11.8
IIIC	14	27.5
Response to therapy *		
Favorable response	48	94.1
Unfavorable response	2	3.9
Median age (range)	34	(22–59)
Median follow-up (range)	21.1	(0.2–39.1)

IGCCCG, International Germ Cell Consensus Classification Group; UICC, Union for International Cancer Control, * in one patient NA.

**Table 2:** Association between the systemic immune–inflammation index and percentage of innate immune cell subpopulations in chemotherapy-naïve GCT patients.

Total White Blood Cell Population (CD45+ Population)	% of Innate Immune Cell Subpopulations						
	Variable	N	Mean	SEM	Median	*p*-Value ^UNI^	*p*-Value ^MVA^
**Total leukocyte subpopulations** (percentage)	**Neutrophil percentage**						
	SII < 1003	32	56.7	1.7	58.7	**0.00000**	**0.00496**
	SII ≥ 1003	19	74.5	2.2	73.8		
	**Monocyte percentage**						
	SII < 1003	32	10.0	0.6	10.2	0.11455	
	SII ≥ 1003	19	8.7	0.7	7.9		
**Monocyte subpopulations** (percentage)	**Classical monocyte percentage**						
	SII < 1003	23	84.9	1.7	85.7	0.12215	
	SII ≥ 1003	17	86.4	2.0	90.7		
	**Intermediate monocyte percentage**						
	SII < 1003	17	5.3	0.7	5.3	0.84080	
	SII ≥ 1003	10	5.1	0.9	5.2		
	**Nonclassical monocyte percentage**						
	SII < 1003	21	5.8	0.9	4.7	0.61768	
	SII ≥ 1003	17	5.2	1.0	5.0		
**Total leukocyte subpopulations** (percentage)	**Polymorphonuclear monocyte (PNMs) percentage**						
	SII < 1003	16	0.2	0.7	0.2	0.72347	
	SII ≥ 1003	14	1.8	0.8	0.2		
	**Eosinophil percentage**						
	SII < 1003	32	3.3	0.4	2.9	**0.00431**	0.63565
	SII ≥ 1003	19	1.4	0.5	1.0		
	**Basophil percentage**						
	SII < 1003	32	0.7	0.1	0.7	**0.00852**	0.92744
	SII ≥ 1003	19	0.5	0.1	0.4		
**Lymphocyte subpopulations** (percentage)	**NKT-cell percentage**						
	SII < 1003	31	2.5	0.5	1.3	0.98405	
	SII ≥ 1003	19	2.6	0.7	1.8		
	**CD4+ NKT-cell percentage**						
	SII < 1003	18	0.3	0.1	0.2	0.24894	
	SII ≥ 1003	10	0.6	0.2	0.3		
	**CD8+ NKT-cell percentage**						
	SII < 1003	19	2.2	0.4	1.1	0.16867	
	SII ≥ 1003	10	2.5	0.6	2.3		
	**NK-cell percentage**						
	SII < 1003	32	10.3	1.6	9.6	**0.02264**	0.06767
	SII ≥ 1003	19	18.3	2.1	15.8		
**Total leukocyte subpopulations** (percentage)	**Dendritic cell (cDCs) percentage**						
	SII < 1003	22	0.9	0.1	0.9	**0.02728**	0.76790
	SII ≥ 1003	14	0.7	0.1	0.6		
	**Plasmocytoid dendritic cell (pDCs) percentage**						
	SII < 1003	22	0.2	0.0	0.2	**0.00310**	0.84143
	SII ≥ 1003	14	0.1	0.0	0.1		
**Subpopulation of DCs** (percentage)	**CD16+ HLADR+ Lin- DC percentage**						
	SII < 1003	13	48.9	5.3	46.1	1.00000	
	SII ≥ 1003	12	46.9	5.5	47.4		
	**CD1c+ within DC percentage**						
	SII < 1003	18	22.5	1.8	20.3	0.02497	
	SII ≥ 1003	15	16.2	2.0	14.7		

UNI—univariate logistic regression analysis; MVA—multivariate logistic regression analysis; significant *p* values are in bold. The variability within the total number of examined patient samples (N) within the evaluated subpopulations was due to the individual technical limitations, including missing antibodies or the poor quality of the examined samples.

**Table 3:** Association between the systemic immune–inflammation index and the percentages of the different adaptive immune cell subpopulations in patients with germ cell tumors.

Total White Blood Cell Population (CD45+ Population)	% of Adaptive Immune Cell Subpopulations						
	Variable	N	Mean	SEM	Median	*p*-Value ^UNI^	*p*-Value ^MVA^
**Total leukocyte subpopulations** (percentage)	**Lymphocyte percentage**						
	SII < 1003	32	31.2	1.5	29.5	**0.00000**	0.42563
	SII ≥ 1003	19	15.0	2.0	13.8		
**Subpopulations of lymphocytes** (percentage)	**B-cell percentage (CD14+)**						
	SII < 1003	32	11.0	0.8	10.7	0.60565	
	SII ≥ 1003	19	10.7	1.0	8.8		
	**T-cell percentage (CD3+)**						
	SII < 1003	32	76.3	1.7	77.3	**0.01410**	**0.01385**
	SII ≥ 1003	19	68.1	2.2	72.3		
	**Helper T-cell percentage**						
	SII < 1003	31	45.7	1.7	47.2	0.37378	
	SII ≥ 1003	19	42.7	2.1	43.8		
	**Cytotoxic T-cell percentage**						
	SII < 1003	32	27.7	1.0	27.9	**0.02383**	0.12797
	SII ≥ 1003	19	23.4	1.3	23.4		
	**T-reg percentage**						
	SII < 1003	32	4.1	0.2	3.8	0.53298	
	SII ≥ 1003	19	3.9	0.3	3.9		

UNI—univariate logistic regression analysis; MVA—multivariate logistic regression analysis; significant *p* values are in bold. The variability within the total number of examined patient samples (N) within the evaluated subpopulations was due to the individual technical limitations, including missing antibodies or the poor quality of the examined samples.

## Data Availability

Data are contained within the article.
